# Nervous Symptoms of Lithæmia

**Published:** 1881-12

**Authors:** 


### NERVOUS SYMPTOMS OF LITHÆMIA.

Few subjects have been of late more studied than that of lithiasis or lithæmia. Able researches have done much to direct medical attention to it, and it is now distinctly known that a state exists which is closely allied to gout, which is characterized by the abundance of lithic acid or lithates in the urine, and frequently coexists with signs of ill-assimilation of food, and with pain unaccompanied by any perceptible changes of the affected part. Hepatic derangement is also often found; and from this end of the chain the links are stretched through many vague symptoms to outbreaks of true gout, or to structural changes in heart, bloodvessels and kidneys. To the obscure nervous symptoms arising from this condition of the blood, Dr. Da Costa has recently directed attention (*American Journal of*

*the Medical Sciences*, October, 1881). One of the most prominent symptoms, according to Da Costa, is vertigo. This, properly speaking, is not the vertigo a stomacho læso of Trousseau, although gastric derangement may be associated with it, having but little connection of a direct character; or one may exist independently of the other, as well as of the more obvious symptoms of lithæmia. In the treatment the correction of the state of the blood is of primary importance. Careful regulation of the diet, reducing both nitrogenous elements and hydrocarbons, forbidding alcoholic drinks, and allowing plenty of water, while systematic exercise, especially in the open air, and due attention to the state of the skin, are all essential. Medicines favoring excretion, purgatives, especially the natural mineral waters, which, at the same time, are diuretic, are to be preferred. Citrate of lithium is particularly serviceable, iodide of potassium and colchicum less so; while remedies, having a direct action upon the nervous system, as Da Costa very correctly points out, should be avoided or used very sparingly, and, as a rule, reserved for special indications.—*Chicago Med. Review.*

